# Brain’s Energy After Stroke: From a Cellular Perspective Toward Behavior

**DOI:** 10.3389/fnint.2022.826728

**Published:** 2022-05-16

**Authors:** Juan José Mariman, Enrique Lorca, Carlo Biancardi, Pablo Burgos, Joel Álvarez-Ruf

**Affiliations:** ^1^Laboratorio de Cognición y Comportamiento Sensoriomotor, Departamento de Kinesiología, Facultad de Artes y Educación Física, Universidad Metropolitana de Ciencias de la Educación, Santiago, Chile; ^2^Departamento de Kinesiología, Facultad de Medicina, Universidad de Chile, Santiago, Chile; ^3^Escuela de Enfermería, Facultad de Medicina, Universidad Finis Terrae, Santiago, Chile; ^4^Biomechanics Lab, Departamento de Ciencias Biológicas, CENUR Litoral Norte, Universidad de la República, Paysandú, Uruguay; ^5^Departamento de Neurociencias, Facultad de Medicina, Universidad de Chile, Santiago, Chile; ^6^Laboratorio de Biomecánica Clínica, Facultad de Medicina Clínica Alemana, Universidad del Desarrollo, Santiago, Chile

**Keywords:** energy, cell damage, locomotion (MeSH), postural control (MeSH), small-world network, stroke (MeSH)

## Abstract

Stroke is a neurological condition that impacts activity performance and quality of life for survivors. While neurological impairments after the event explain the performance of patients in specific activities, the origin of such impairments has traditionally been explained as a consequence of structural and functional damage to the nervous system. However, there are important mechanisms related to energy efficiency (trade-off between biological functions and energy consumption) at different levels that can be related to these impairments and restrictions: first, at the neuronal level, where the availability of energy resources is the initial cause of the event, as well as determines the possibilities of spontaneous recovery. Second, at the level of neural networks, where the “small world” operation of the network is compromised after the stroke, implicating a high energetic cost and inefficiency in the information transfer, which is related to the neurological recovery and clinical status. Finally, at the behavioral level, the performance limitations are related to the highest cost of energy or augmented energy expenditure during the tasks to maintain the stability of the segment, system, body, and finally, the behavior of the patients. In other words, the postural homeostasis. In this way, we intend to provide a synthetic vision of the energy impact of stroke, from the particularities of the operation of the nervous system, its implications, as one of the determinant factors in the possibilities of neurological, functional, and behavioral recovery of our patients.

## Introduction

Stroke is a leading cause of death and disability in many Western nations (Coupland et al., 2017). The main alteration after stroke is motor impairment, which affects the control of face, arm, and leg movements and is present in about 80% of patients (Walker et al., 2017). More than 30% of survivors still cannot walk independently at 6 months (Corbetta et al., 2015). These deficits are associated with specific impairments in the upper (UL) and lower limb (LL). It is estimated that alterations of UL function occur in 85% of patients during the first days after the stroke (Koh et al., 2015), and the sequelae, after 6 months, persist between 55 and 75% (Buma et al., 2013). Similar figures were reported for LL (Aqueveque et al., 2017).

Motor impairments of LL and UL cause limited use or non-use in activities of daily living (ADL), such as dressing, cooking, or bathing (Nichols-Larsen et al., 2005; Price and Choy, 2019). Therefore improving UL functions for reaching and manipulating objects is a central element in post-stroke rehabilitation, which requires the complex integration of neuromuscular activity from the trunk to the fingers (Dobkin, 2005). Complementarily, improving the postural control and LL functions during gaits is crucial for performing functional and social activities (Pollock et al., 2007).

**FIGURE 1:**
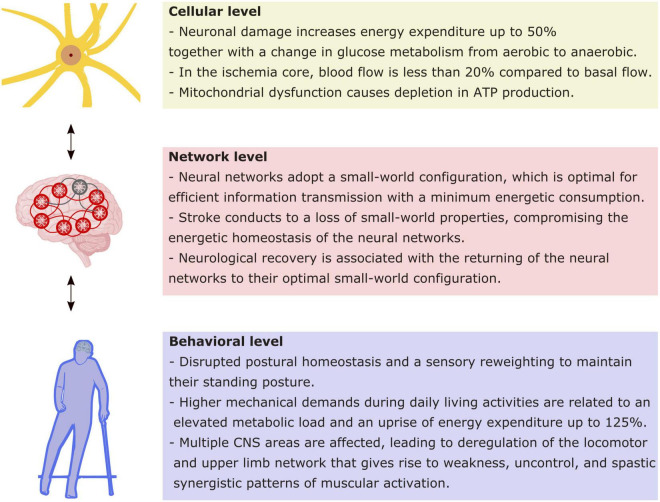
Energy implications at different levels of functioning as a consequence of stroke. Schematic synthesis of the main implications of stroke in energetic functioning at the cellular, network, and behavioral levels. Every level impacts reciprocally in the following, as indicated by bidirectional arrows.

Studies have shown that there is an early window of cerebral plasticity post-stroke given by the injury physiopathology (Ng et al., 2015; Chippala and Sharma, 2016; Zeiler et al., 2016). One question regarding this early window is how to perform rehabilitation to achieve the greatest functionality with the least compensation. Such a challenge requires a broad and multisystemic view of the impairment associated with the stroke that must cover from cellular to behavioral level. Current theoretical approaches have highlighted the role of energy homeostasis as a constraint for the operation of the nervous system. From this point of view, we will summarize the current evidence at three levels of nervous system organization to describe the energy implication of their functioning, both in healthy and after a stroke (Figure 1).

## Energy and Cell Damage

Generally, when we talk about energy processes in mammalian cells, we refer to the metabolic pathways associated with ATP production linked to the cyclization of NADH (Rigoulet et al., 2020). Our cells use bioenergetic molecules to carry out processes such as metabolism regulation, transport across membranes, biosynthesis of new molecules, and mechanical energy generation (Atkinson, 1977; Suarez, 2012). However, these processes depend directly on two fundamental factors: (1) mitochondrial integrity and (2) oxygen availability (Nunnari and Suomalainen, 2012; Rigoulet et al., 2020). These factors are fundamental in that they can promote neuronal processes such as synaptic plasticity in mature and aging nervous systems (Todorova and Blokland, 2016). Therefore, if we consider the stroke as an event in which the blood flow to specific regions of our brain is interrupted (Sacco et al., 2013; Campbell et al., 2019), it is logical to bear in mind that the energy metabolism of the affected areas has significant alterations.

General mechanisms in cell damage are characterized by multiple physiological responses that involve increased energy expenditure, increased lipid and protein catabolism, nitrogen imbalance, hyperglycemia, among others (Kinney, 1995; Chioléro et al., 1997). It has been determined that the basal metabolic rate of subjects who have undergone a surgical procedure or who have suffered brain trauma, increases between 20 and 50% (Weissman and Kemper, 1992; Petersen et al., 1993; Chioléro et al., 1997). Likewise, during cerebral ischemia, glucose metabolism changes significantly, passing from aerobic metabolism to anaerobic one (Schurr, 2002) and activating the pentose phosphate cycle, which increases the ratio between NADPH/NAD+ (Sahni et al., 2018) and therefore, an increase in metabolic rate.

Strokes are characterized by an impairment of the blood, nutrients, and oxygen supply, to brain tissue (Campbell et al., 2019). Likewise, stroke areas were classified into: (1) the part of the brain with blood hypoperfusion, changes in metabolism, and electrical hypoactivity were called the ischemic core, and (2) the region with progressive loss of functional tissue was called the penumbra zone (Astrup et al., 1981; Campbell et al., 2019). In the ischemic core, blood flow is less than 20% compared to basal flow (Lo, 2008), which results in immediate oxygen and glucose deprivation, causing neurons to be unable to produce electrochemical gradients, in addition to increasing the intracellular calcium concentration, which is cytotoxic in large quantities (Iadecola and Anrather, 2011; Marlier et al., 2015). Otherwise, the region of the ischemic penumbra has a partially compensated blood flow, so it is not exposed to energetic disorders related to the ischemic core, however, these neurons are exposed to glutamate accumulates in the affected region (Iadecola and Anrather, 2011; Marlier et al., 2015).

Mitochondrial dysfunction after a stroke contributes to an increase in reactive oxygen substances and a depletion in ATP production (Semple, 2014). Furthermore, the alteration of mitochondrial metabolism will result in the activation of cellular pathways that promote cell death processes (Kasahara and Scorrano, 2014; Datta et al., 2020). Likewise, oxygen metabolism is significantly reduced, considering that factors such as blood perfusion, cerebral blood flow, and cerebrovascular resistance are in homeostatic unstable states, directly affecting mitochondrial integrity (Lin and Powers, 2018). Consequently, alterations in brain metabolism mediated by stroke result in a significant energy depletion in the affected tissue, which contributes to the loss of functional cells, the decrease of the molecules that participate in the basal metabolic processes, and global alterations in mitochondrial and oxygen metabolism.

The metabolic alterations caused by the stroke not only bring consequences to the regions directly involved but also affect other organism processes, such as abnormal activation of the muscles (Macko et al., 1997; Hunnicutt and Gregory, 2017), changes in the metabolism of amino acids and lipids (Wang et al., 2020), elimination of reactive substances oxygen and DNA damage (Li P. et al., 2018), among others. For example, some compilation studies have identified many metabolites involved in multiple metabolic pathways that change their levels significantly around the stroke episode (Au, 2018; Shin et al., 2020).

The progressive loss of functional brain tissue due to ischemia leads to alterations in the general functionality of the neuronal networks and changes in the basal metabolism of the affected people. However, the endogenous mechanisms of tissue repair and the initiation of neurogenesis are significantly impaired (Toman et al., 2019) by ongoing inflammatory processes and DNA damage (Li P. et al., 2018). Until now, few studies have conclusive results related to the recovery of damaged tissue, however, some approaches shed light on which guidelines to follow to promote the recovery of metabolism after a stroke (Kuriakose and Xiao, 2020). It has been observed that HIF-1α, a protein that participates as an intermediary in cell adaptation in response to hypoxia, could be an excellent therapeutic target in stroke since it favors the reactivation of neural stem and progenitor cells and local angiogenesis (Cunningham et al., 2012). This is how new pharmacological targets have been explored to promote neuronal repair (Wu et al., 2017) and re-stabilize the lost energy metabolism.

## Energy and Neural Networks

Neural communication is the most expensive operation in the nervous system. Due to the high flow of information, energetic consumption of the nervous system reaches ∼20% of the total energy consumption of the body at resting state, even though it only represents 2% of the body mass (Shulman et al., 2004). This consumption increases in regions identified as hubs for the network, as proved by correlation studies between functional connectivity and cerebral blood flow, a surrogate of cerebral metabolism. The majority of energetic expenditure is associated with signal transmission, which depends on active processes for maintaining several events, including the ionic gradient for action potential propagation and pre and postsynaptic events for network communication (Yu and Yu, 2017). Consequently, a decrease in the energetic supply interrupts the operation of neural networks. A paradigmatic case is a stroke, where a drop in blood flow by an arterial conflict (thrombosis, embolism, or hemorrhage) provokes a deterioration of the functioning of brain regions dependent on such a source of energy. The extreme energy dependence of brain tissue is manifested by the fact that initial neurologic impairments could be reverted if reperfusion strategies restitute the blood flow (Imran et al., 2021). This brain pathophysiology supports current theoretical proposals that highlight the role of energy homeostasis as a constraint for neuronal processes at the molecular, network, and behavioral levels (Vergara et al., 2019). At the network level, the energy homeostasis principle sets that individual neuron activity influences the network activity to reach a homeostatic state between production and energy consumption. Such interdependency for the network components exhibits an optimal topology that can be described by theoretical graph analysis. Thus, complex networks are characterized by an optimal configuration where every component or “node” is connected with their neighbor by the shortest connection, forming local clusters but including few long connections for distant nodes (Watts and Strogatz, 1998). This configuration, named “small-world,” guarantees a shorter path between nodes, optimizing the connectivity, leading to an optimal timing for signal transmission with a reduction in wiring (axons), minimizing the energy cost (Bassett and Bullmore, 2006). Long brain connections impose a high energy cost associated with sodium conductance (Yu and Yu, 2017). Therefore, a small-world setting is an appropriate tradeoff between optimal communication and energy consumption, reaching a homeostatic state. This feature is especially relevant to maintain a large-scale distributed information processing, which has been proposed as the brain’s functional organization that supports sensorimotor and cognitive control of behavior (Varela et al., 2001).

What happens in stroke, where network components are dysfunctioning (penumbra region) or lost (core region)? Neuroimaging technics allow the description of network reorganización after a stroke. The majority of recent studies report the conservation of small-world definition for the network state, but with a deterioration of specific small-world indices in acute (Caliandro et al., 2017; Shi et al., 2021) and subacute stages of the stroke (Wang et al., 2010; Zhang et al., 2017; Li et al., 2021). Interestingly, the preservation (Bonilha et al., 2016) and recovery (Caliandro et al., 2017; Li et al., 2021) of optimal small-world properties of the network correlate positively with the improvement of cognitive and motor functions, suggesting that small-world indices restoration could be a proxy for predicting neurological recovery (Vecchio et al., 2019). Studies in animal models (van Meer et al., 2012) have complemented these findings, showing that the initial small-worldness (an excessive clustering and wiring of the network) evolves to an optimal small-world topology in the chronic stage. Such network remodeling is accompanied by a recovery of the disrupted corticospinal system in parallel to the improvement in sensorimotor performance, which is similar to the association between corticospinal excitability and hand motor recovery described in humans (Veldema et al., 2018). As discussed previously, a small-world configuration is an energy-efficient communication model compared to random networks (Zhang et al., 2013); therefore, stroke initially leads to a loss of optimal network configuration, with a consequent energy inefficiency neuronal functioning. Consistently, neurological recovery is associated with a network reorganization that shows better small-world indices, and therefore, energy optimization. This optimization at the network level impacts behavior, where the recruitment of the corticospinal descending system allows an efficient and effortless upper limb performance (representing an optimal network functioning). In comparison, the compensatory use of alternative descending systems (as the rubro and reticular spinal tract or the ipsilesional corticospinal tract, all suboptimal networks) provoke effortful and inaccurate hand control with a high energy cost for movement (Takenobu et al., 2014; Jones, 2017). Future research incorporating energetic measures associated with network analysis could give us a complete description of network dynamics behind stroke recovery.

## Energy and Motor Behavior

### Implications for Postural Control

Movement is probably one of the principal manifestations of the nervous system, with three principal characteristics: coordinated, propositive, and adaptative to the ambient’s demands. In this specific scenario, postural control (PC) arises as a fundamental behavior. In a daily living scenario, the system has to deal with these conditions to maintain the adaptability of posture with the variability good enough to be stable against any demands (task and ambient). The expertise in the perception-action couple would influence the person’s performance (Seifert et al., 2014). In pathological conditions, postural homeostasis might be disrupted. After a stroke, the patients have deficits in PC (Tasseel-Ponche et al., 2015) and a sensory reweighting to maintain their standing posture (Bonan et al., 2013).

With the help of posturography, has been reported a decrease in PC when it is evaluated in eyes close condition and a dual-task condition, and an altered bodyweight distribution compared with healthy individuals (Bensoussan et al., 2007).

Stroke causes several physical impairments, that limit the functionality of the patients. Fatigue is one of them and is related to an elevated metabolic load due to higher mechanical demands during daily living activities. Houdijk et al. (2010) reported no differences in basal metabolism compared with healthy subjects, but when the comparison is made during upright position, the differences are significant, where the net average energy expenditure was 125% higher for patients with stroke (Houdijk et al., 2010).

### Implications for Locomotion

Locomotion is a complex motor function controlled by a Central Pattern Generator (CPG) located in the spinal cord (Guertin, 2009). The modular organization of muscle synergies, the source of the basic rhythmic limbs movements, is “embedded” in the CPG (Tresch et al., 1999), goal-directed locomotion needs posture and steering control systems, which are scattered in different CNS areas, like the cortex, basal ganglia, cerebellum, brainstem (Grillner et al., 2008; Guertin, 2009). Therefore, locomotion relies on efficient communications through ascending and descending pathways, to assure sensory-motor integration.

As we have reviewed previously, neural network dynamic is altered from energy failure associated with stroke. In the case of locomotion, damage of the motor cortex and its output, the corticospinal tract, translates into deregulation of the locomotion network at the level of descending subcortical pathways and spinal circuits, giving rise to weakness, spasticity, and spastic synergistic patterns of muscular activation (Li S. et al., 2018). These impairments explain the stereotypical hemiplegic gait, which has a poor biomechanical performance despite its elevated energy cost.

Lower limb muscles during walking are organized in four synergy modules, corresponding to specific parts of the stance (weight acceptance, midstance, and toe-off) and swing phases (Pequera et al., 2021). However, in post-stroke patients, a reduction of the number of synergy modules in the paretic limb has been observed, and most of them only require two or three modules (Clark et al., 2010). Such reduction, a consequence of changes in the neural communication pathways (Clark et al., 2010), leads to a higher level of co-contractions and slower and less fluid movements, which results in a reduced self-selected walking speed (SSWS) (Detrembleur et al., 2003).

Therefore, the stroke would cause a cascade of events, started in the CNS, which affect the motor coordination and the mechanics of the paretic limb. Its poor contribution to the push-off would determine a compensatory action of the non-paretic limb (Chen et al., 2005; Farris et al., 2015). This includes an increased circumduction of the paretic leg during the swing, also resulting in a counteracting higher torque of the other leg (Shorter et al., 2017). Despite such asymmetries, the pendular mechanism of walking is maintained (Zamparo et al., 1995), but with limited potential and kinetic energy interchanges, due to reduced amplitude of the kinetic energy. This would reduce the energy recovery and increase the external mechanical work (Detrembleur et al., 2003).

The motor coordination and mechanical impairments determine, in post-stroke patients, a high metabolic cost of walking per unit distance (C) (Zamparo et al., 1995). When provided with handrail supports, patients improve both their SSWS and the C, suggesting that their extra cost is partially due to enhanced efforts for balance control (Ijmker et al., 2013). The low SSWS itself contributes to increasing the C, being located within the left rising branch of the “U” shaped C versus speed relationship, as frequently observed in pathological walking gaits (Bona et al., 2017, 2020).

### Implications for the Upper Limb Control

After a stroke, the damage of neuronal and glial tissue produces a loss of selectivity and efficiency of the control of the upper limb (Murphy and Corbett, 2009). Usually, this damage is compensated with alternative neural networks, alternative kinematic patterns, and greater cognitive control which at the brain and body level implies a greater energy expenditure (Cirstea and Levin, 2000; Levin et al., 2002, 2009; Jones, 2017; Balbinot et al., 2022).

This damage to the cortical control network could disrupt a small-world organization, which has an impact on energy efficiency and the selective and flexible control (Gerloff and Hallett, 2010; Rehme and Grefkes, 2013; Li et al., 2021).

## Conclusion

Based on the literature available, we have discussed the effect of stroke, going from cellular to behavioral levels.

In stroke patients the cellular mechanisms are disrupted, affecting not only the neural network functioning but also postural and locomotion control. The impairment of blood, nutrients, and oxygen generates a chain reaction, conducting to a hyper-reactive state and mitochondrial dysfunctions, leading to metabolic dysfunction, causing an energetic lack. At the network level, stroke leads to a loss of small-world topology, which compromises the information processing and leaves the brain network in an energy inefficient operating state. Fortunately, post-stroke recovery is accompanied by a variable degree of restoration of small-world properties, which is associated with neurological recovery. Thus, the stroke compromises the energy homeostasis of the network. At a behavioral level, upper limb, posture and gait are frequently affected, provoking several dysfunctions and functional limitations. Such motor impairments could be classified into the category of stability problems, causing poor control of the inherent variability of the motor behavior. These stability problems lead to over-expanding energy to compensate for the reduction of synergies involved in the motor control.

Finally, recent changes in the paradigms of studying the nervous systems that incorporate the energy in the formula, represent a big step forward to a multifactorial and integrative comprehension of the functioning of the nervous system in healthy and impaired conditions.

## Data Availability Statement

The original contributions presented in the study are included in the article/supplementary material, further inquiries can be directed to the corresponding author.

## Author Contributions

JM, EL, CB, PB, and JÁ-R contributed equally to the bibliographic research, selection and synthesis of information, manuscript redaction, and approved the submitted version.

## Conflict of Interest

The authors declare that the research was conducted in the absence of any commercial or financial relationships that could be construed as a potential conflict of interest.

## Publisher’s Note

All claims expressed in this article are solely those of the authors and do not necessarily represent those of their affiliated organizations, or those of the publisher, the editors and the reviewers. Any product that may be evaluated in this article, or claim that may be made by its manufacturer, is not guaranteed or endorsed by the publisher.
